# The Predicted Impact of Ipilimumab Usage on Survival in Previously Treated Advanced or Metastatic Melanoma in the UK

**DOI:** 10.1371/journal.pone.0145524

**Published:** 2015-12-23

**Authors:** James Larkin, Anthony J. Hatswell, Paul Nathan, Maximilian Lebmeier, Dawn Lee

**Affiliations:** 1 The Royal Marsden Foundation NHS Trust, Fulham Rd, London, SW3 6JJ, United Kingdom; 2 BresMed, 84 Queen St, Sheffield, S1 2DW, United Kingdom; 3 Mount Vernon Cancer Centre, Rickmansworth Rd, Northwood, Middlesex, HA6 2RN, United Kingdom; 4 Bristol–Myers Squibb Pharmaceuticals Limited, Sanderson Road, Uxbridge, Middlesex, UB8 1DH, United Kingdom; University of Queensland Diamantina Institute, AUSTRALIA

## Abstract

**Background:**

Evaluating long-term prognosis is important for physicians, patients and payers. This study reports the results of a model developed to predict long-term survival for UK patients receiving second-line ipilimumab.

**Methods:**

MDX010-20 trial data were used to predict survival for ipilimumab versus UK best supportive care. Two aspects of this analysis required novel approaches: 1) The overall survival Kaplan–Meier data shape is unusual: an initial steep decline is observed before a ‘plateau’. 2) The need to extrapolate beyond the trial end (4.6 years). Based upon UK clinician advice, a three-part curve fit was used: from 0–1.5 years, Kaplan–Meier data from the trial; from 1.5–5 years, standard parametric curve fits; after 5 years, long-term data from the American Joint Committee on Cancer registry.

**Results:**

This approach provided good internal validity: low mean absolute error and good match to median and mean trial data. Lifetime predicted means were 2.77 years for ipilimumab and 1.07 for best supportive care, driven by increased long-term survival with ipilimumab.

**Conclusion:**

To understand the full benefit of treatment and to meet reimbursement requirements, accurate estimation of treatment benefit is key. Models, such as the one presented, can be used to extrapolate beyond trials.

## Background

Melanoma is an aggressive form of skin cancer with a rising incidence in the UK, which is currently approximately 17 per 100,000 [1]. Although melanoma represents only 4% of all skin cancer cases, because of its aggressive nature, it accounts for 80% of all skin cancer deaths [2]. Malignant melanoma has an unusual pattern compared with most other cancer sites in that many patients are diagnosed at a young age. In the UK, between 2008 and 2010, an average of 27% of cases were diagnosed in those aged under 50 years, and an average of 45% of cases were diagnosed in the 65s and over [1].

If detected before it has spread, melanoma can be cured by surgical excision. Malignant melanoma is the fifth most common cancer in the UK, but only the 18th most common cause of cancer death, reflecting high survival from the disease [1]. Whilst prognosis has improved markedly in recent decades and is good for early stage disease treated with adequate surgery, once metastasis has occurred, prognosis is poor: for Stage IV melanoma, historically median survival has been approximately 6–9 months [1, 3, 4].

Until 2012, dacarbazine was the only UK recommended treatment option for unresectable Stage III (regional lymph nodes involved) and Stage IV (metastatic) disease. There was no standard second-line treatment. Oncologists either entered patients into clinical trials, provided only supportive therapies, or administered a variety of off-label systemic chemotherapies–all with palliative intent [5, 6]. None of these agents demonstrated a survival benefit in clinical trials, and all are associated with significant toxicity [7, 8]. The UK pathway of care for melanoma has changed considerably following the licensing and recommendation by the National Institute of Health and Care Excellence (NICE), between 2012–2014, of the immunotherapy ipilimumab, and the BRAF inhibitors vemurafenib and dabrafenib, which can be used in the 50% of patients whose melanoma harbours an activating mutation in the BRAF gene [8–11]. First-line standard of care outside of clinical trials is currently stratified by tumour and patient characteristics, taking into account the presence or absence of BRAF mutation, with recently published literature on treatment sequencing, supporting the sequencing of immunotherapy prior to BRAF inhibitors for less aggressive tumours [12, 13]. Chemotherapy and supportive therapies remain the only option for patients who have received ipilimumab and BRAF inhibitors where appropriate.

In 2012 NICE recommended ipilimumab (Yervoy^®^), a fully human monoclonal immunoglobulin antibody (IgG1κ), for use as a second-line treatment based upon evidence from the MDX010-20 clinical trial (Clinicaltrials.gov: NCT00094653) [14]. The effectiveness of ipilimumab at the licensed 3mg/kg dose was studied in this 56-month, double-blind, controlled study, in which patients were randomised to ipilimumab+gp100, ipilimumab alone or gp100 alone. Gp100 is an experimental vaccine that had been shown to increase the effectiveness of IL-2 immunotherapy; however, it has limited anti-tumour activity as a single agent [15]. Both ipilimumab-containing arms showed statistically significant benefits in overall survival when compared to gp100 monotherapy (median of 10.0 vs 10.1 vs 6.4 months). Longer-term data is available for ipilimumab from a pooled analysis of patients treated at different doses and regimens, demonstrating a sustained survival benefit for a proportion of patients for up to 10 years [16, 17].

Ipilimumab offers a novel way to stimulate the body’s own immune system to fight cancer. When the immune system detects a foreign antigen (in this context, the tumour), an immune response is launched, and the foreign antigen is attacked by T-cells. This response is self-limiting and controlled through a number of checkpoints including CTLA-4 (cytotoxic T-lymphocyte antigen 4). By blocking CTLA-4 activity, ipilimumab reduces the immune response from being switched off, allowing the number and production of active T-cells to increase in order to target the tumour more effectively. Ipilimumab’s mechanism of action is therefore fundamentally different from other conventional melanoma (and cancer) treatments (including BRAF inhibitors), which is reflected in the unusual survival profile discussed in this paper [18, 19].

An understanding of the long-term prognosis following treatment with ipilimumab is not only important for physicians and patients but also for payers who evaluate the likely long-term effectiveness and cost-effectiveness of different treatment strategies to inform reimbursement decisions. The health-economic justification of a treatment from which only a minority of patients benefit, but for whom the benefits are long-lasting, depends upon being able to model the long-term benefits of treatment.

This study reports the results of a model developed to predict the long-term survival of patients treated in the UK with second-line ipilimumab, which was used within NICE’s appraisal of ipilimumab as a second-line treatment option [9].

## Methods

To assess the cost-effectiveness of ipilimumab as a second-line treatment, a semi-Markov model was constructed in Microsoft Excel 2010 (Microsoft Ltd, Redmond, WA, USA). In line with NICE Decision Support Unit guidance, parametric curve fits were used to describe the survival of patients treated with ipilimumab and the most relevant UK comparator–best supportive care (BSC), which was defined as a mixture of chemotherapy and supportive therapy [20]. The model was used to calculate estimates of the mean and median life years for an average patient and the estimated survival at various time points of interest: 1 year, 2 years, 5 years, 10 years and 20 years. Additionally, conditional survival probabilities were calculated for long-term survivors (i.e. patients still alive at Year 2) to predict the probability of survival for up to 25 years.

### Trial data

Among 676 patients enrolled in MDX010-20, 403 were randomly assigned to receive ipilimumab plus gp100, 137 to receive ipilimumab alone, and 136 to receive gp100 alone (the control group). Included among these patients were 82 (12.1%) patients who had metastases in the central nervous system at baseline. The mean age of the patients included in the trial was 56.2, the majority of patients had an Eastern Co-operative Oncology Group (ECOG) performance status of 0 (55.3%) or 1 (43%) and had lactate dehydrogenase (LDH) within the normal range (61.7%) [14]. Furthermore, 656 patients (98.2%) had Stage IV melanoma, and 483 (71.4%) had Stage M1c disease.

As no drug has been previously shown to provide a benefit in either progression-free or overall survival for metastatic melanoma patients, the data from the gp100 group in MDX010-20 was used as a proxy for BSC in the model. Gp100 has a similar impact on survival to placebo treatment and the treatments used for pre-treated advanced melanoma before the introduction of ipilimumab (dacarbazine, paclitaxel and carboplatin) [14, 21].

In MDX010-20, there was no significant difference between the two arms where patients received ipilimumab, and no noticeable effect was observed with the addition of gp100 to ipilimumab alone (median survival 0.83 vs 0.84 years). Therefore, the datasets for ipilimumab monotherapy (n = 137) and ipilimumab+gp100 (n = 403) were considered to be combinable, which would increase the sample size available for analysis (n = 540).

### Methods used to predict long-term survival

In performing this analysis, two aspects required advanced modelling approaches.

**Fig 1:**
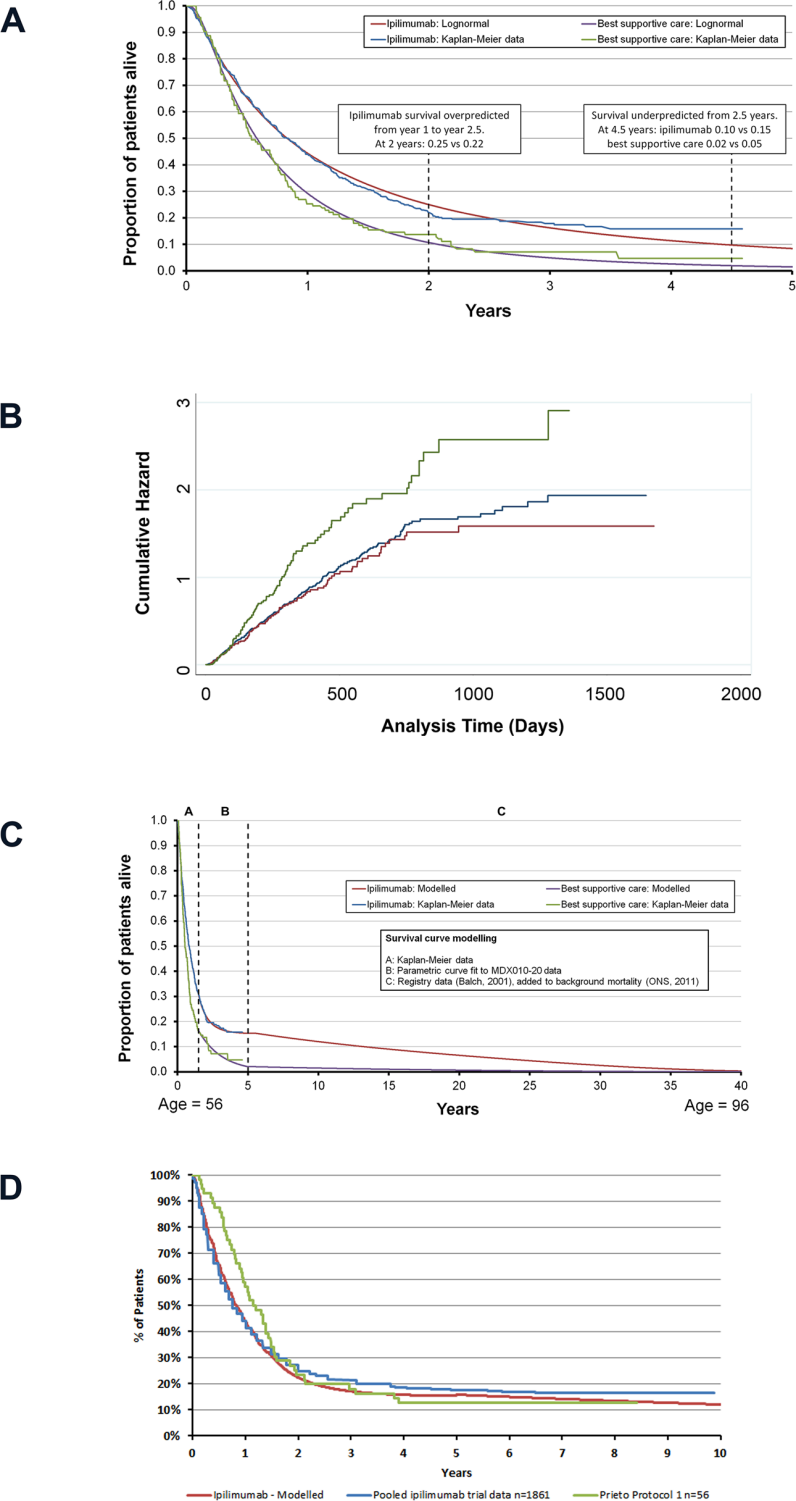
A: Parametric curve fits applied to MDX010-20 data; B: Cumulative hazard plot for ipilimumab vs ipilimumab + gp100 vs gp100; C: Estimated survival curves using three-part curve fit; D: Comparison of modelled survival estimates with available trial data.

Firstly, the shape of the overall survival Kaplan–Meier data in MDX010-20 is unusual (Fig 1A) as an initial steep decline is observed before the curve reaches a ‘plateau’. The plateau extends from approximately 1.5 years until the end of the trial (4.6 years) and represents long-term survival benefit for a proportion of patients. Longer-term data from pooled analyses confirm that this plateau is maintained for up to 10 years in some patients [14, 16, 17]. This survival profile is to be expected for ipilimumab, which is likely due to its mechanism of action as an immunotherapy; ipilimumab requires the majority of the 4 dose course to be received and time following therapy to exert its (indirect) anti-tumour immune effects [13].

Secondly, there is the need for extrapolation beyond the end of the trial period as, although the trial period is long (4.6 years), approximately 17% of patients treated with ipilimumab were alive at the end of the trial compared with 4% in the BSC arm. To compare the means, the survival profile of these patients had to be extrapolated.

There are a number of methods available for performing extrapolation. Exponential, Weibull, Gompertz, log-logistic or log-normal parametric models are commonly used, with each having unique characteristics that make them suitable for different data sets. All of these models make assumptions regarding the rate of events (deaths) at time t, conditional upon survival until time t; this is known as the hazard function [20]. However, none of these models allow the assumption of a high hazard of death initially, with a low hazard for longer-term survivors, as is observed in the ipilimumab datasets. As a result, when these standard functions were plotted against the observed Kaplan–Meier data from MDX010-20, they fitted poorly. Fig 1A shows the best fitting of these standard functions according to the Akaike Information Criterion (AIC) statistical goodness of fit test; the log-normal curve. Using this standard approach overestimated ipilimumab survival between 1 and 2.5 years but under-estimated survival after 2.5 years. Table 1 shows the mean absolute errors (MAEs) associated with each of the different potential curve fits for ipilimumab and BSC. The MAEs are relatively high and even higher for patients who survived more than 1.5 years, with prediction errors of 2–6%. The challenge of estimating survival based on this dataset was presented at ISPOR Europe 2011 and was covered by Annemans *et al* [22].

**Table 1:** Mean absolute error of curve-fitting approaches to MDX010-20 overall survival trial data.

Curve Fit	Ipilimumab		Gp100*	
	MAE	MAE 1.5 year survivors^+^	MAE	MAE 1.5 year survivors^+^
**Parametric curve fit (not used in the final model)**				
Weibull	0.047	0.054	0.039	0.033
Log-normal	0.022	0.029	0.018	0.019
Log-logistic	0.021	0.029	0.015	0.017
Exponential	0.055	0.062	0.034	0.028
Gompertz	0.019	0.021	0.025	0.017
**Three part curve fit** * **, i.e. Kaplan–Meier data to 1.5 years then parametric curve fit until year 5 followed by registry data (used in the final model)**				
Weibull	0.010	0.014	0.008	0.012
Log-normal	0.008	0.011	0.005	0.008
Log-logistic	0.009	0.014	0.006	0.009
Exponential	0.019	0.028	0.008	0.012
Gompertz	0.003	0.004	0.007	0.011

MAE, mean absolute error.

* gp100 is used as a proxy for the efficacy of UK best supportive care

^+^ the third part of the curve fit (registry data beyond the end of the trial period) was not required for these comparisons, which are conducted only over the duration of the MDX010-20 trial.

One alternative approach to modelling survival for similar datasets is mixture survival analysis (or responder based analysis). In the case of the MDX010-20 dataset, however, there are difficulties implementing this type of approach as response was only measured at two timepoints using RECIST criteria. Further, as patterns of response with cancer immunotherapy differ from those with cytotoxic chemotherapy, some immunotherapy-responders may incorrectly be classed as progressors due to tumour flare upon response.

As a result of the difficulties in fitting parametric curves to the data (shown in Fig 1A) and unsuitability of responder based analysis, the Nelson–Aalen cumulative hazard estimate was plotted (Fig 1B), which showed the hazard rate for ipilimumab changing at around 1.5 years (4.6 years of data were available). To model the survival curve more accurately, the curve was split into three sections: from 0 to 1.5 years; from 1.5 to 5 years (reflective of the duration of the clinical trial); and more than 5 years (beyond the end of the clinical trial).

In the initial period from 0 to 1.5 years, standard parametric curve fits failed to provide a good fit to the data (owing to the steep falls observed in the initial stages). For this reason, the Kaplan–Meier data from the trial was included directly with no curve fitting. For the period from 1.5 to 5 years, standard parametric curve fits were employed [20], with the best-fitting curves selected through the use of the AIC. For the ipilimumab survival data, the best-fitting curve could be produced by means of a Gompertz function, and for BSC, the best-fitting curve was produced by an Exponential function.

Trial data were available for patients who survived less than 5 years. To supplement this, data from the AJCC registry reported by Balch *et al* were used [4]. This paper provided melanoma-specific mortality (by stage) up to 15 years and was used to provide long-term survival estimates. Data were included from the study by Balch *et al* for 1,158 patients with Stage IV melanoma (in MDX010-20, 98.2% had Stage IV melanoma). The registry mortality rates were added to the background mortality rates of the UK population aged 56 (the mean age on entry to the study), taken from the Office of National Statistics [23].

This method was employed following consultation with UK clinicians, who identified it as the most robust approach to estimate long-term survival for ipilimumab.

### Validation

It is important to attempt to validate the predictions made by survival models both internally (fit to the trial data) and externally (comparison to alternative datasets and clinical expectation). Internal validity was tested using the MAE of the fitted curves to observed data and through visual inspection of the predicted versus observed curve fits.

External validity of the survival curves was tested both through validation against the expectations of four practising UK oncologists, independently, via visual presentation, with questions and clarifications encouraged, and through presentation to six economic experts at an advisory board. The survival projections were compared to published long-term survival data for ipilimumab from Prieto *et al* (56 patients from a clinical trial of previously treated patients) and the recently published pooled analysis of 1,861 patients over all lines of therapy reported by Schadendorf *et al*, which was not available at the time of analysis [16, 17].

## Results

The use of the three-part curve fit approach provided good internal validity, significantly reducing the MAE associated with the curve fits (Table 1). The curves selected for overall survival have a MAE of only 0.003 (0.004 for patients who survived beyond 1.5 years) in the ipilimumab arm and 0.008 (0.012 for patients who survived beyond 1.5 years) in the BSC arm. The resulting survival curves are shown in Fig 1C.

The predicted median and mean survival observed within the trial (4.6 years) matches well with the modelled values (Table 2), with a modelled and observed median of 0.83 years for ipilimumab versus 0.53 for BSC. Lifetime predicted means were 2.77 years for ipilimumab and 1.07 for BSC. The majority of the difference in lifetime survival is driven by the increased proportion of patients in the ipilimumab arm shown to experience long-term survival (Fig 1C).

**Table 2:** Model-predicted survival vs. survival observed in the clinical trial.

Survival (years)	Trial: ipi alone	Trial: ipi+gp100	Trial: combined ipi arms	Model: ipi	Trial: gp100^+^	Model: BSC
Median	0.84	0.83	0.83	0.83	0.53	0.53
Restricted Mean^+^	1.55	1.40	1.45	1.35	0.90	0.91
Lifetime Mean	n/a	n/a	n/a	2.77	n/a	1.07
**Proportion of patients alive (%)**						
1 year	45.6	43.6	44.1	44.1	25.3	25.3
2 years	23.5	21.6	22.1	22.5	13.7	12.1
3 years	20.1	17.0	17.8	17.1	7.1	6.7
4 years	20.1	14.2	15.8	15.7	4.8	3.7
5 years	n/a	n/a	n/a	15.3	n/a	2.0
10 years	n/a			12.0		1.5
15 years	n/a			9.0		1.0
20 years	n/a			6.6		0.7
25 years	n/a			4.4		0.4

BSC, best supportive care; ipi, ipilimumab; n/a, not applicable.

* gp100 is used as a proxy for the efficacy of UK best supportive care

+restricted to the maximum observed trial value (4.6 years)

For patients who survived 2 years after ipilimumab treatment, the estimated overall survival at 5 years is 67% (Table 3), at which point the model uses only trial data. Following this, point estimates are based on registry data but show high levels of overall survival (estimated as 54% at 10 years post treatment and as 4% at 25 years). The median overall survival for long-term survivors–patients who survived for at least 2 years–was approximately 12 years, (by which point patients would be aged 70 using baseline characteristics from MDX010-20). The relatively tight confidence intervals surrounding the estimates should be noted. However, this is conditional on the historical registry data being appropriate for patients treated with ipilimumab. In BSC long-term survivors, the prognosis is poorer, with only 1.7% expected to survive 10 years and with all patients expected to have died by 25 years. This underlines the difference in survival between the two arms.

**Table 3:** Conditional survival probabilities, i.e. probability (%) of being alive, given that the patient was alive at year 2.

Year	Ipilimumab		BSC/Gp100*	
	Modelled results, % (95% CI)	Trial results, % (95% CI)	Modelled results, % (95% CI)	Trial results, % (95% CI)
2	100	100	100	100
3	75.8 (67.1, 82.8)	78.5 (67.1, 91.7)	46.2 (37.9, 53.6)	51.9 (31.3, 86.2)
4	69.2 (55.1, 79.7)	65.8 (50.9, 85.0)	24.7 (17.0, 32.1)	n.e.
5	67.0 (48.7, 79.1)	n.e.	14.5 (8.6, 20.7)	n.e.
10	53.5 (38.9, 63.2)	n.e.	1.7 (0.5, 3.3)	n.e.
15	40.3 (29.3, 47.5)	n.e.	0.3 (0.1, 0.8)	n.e.
20	29.3 (21.3, 34.6)	n.e.	0.1 (0.0, 0.3)	n.e.
25	19.8 (14.4, 23.3)	n.e.	0.0 (0.0, 0.1)	n.e.

BSC, best supportive care; CI, confidence interval; n.e., non-evaluable.

* gp100 is used as a proxy for the efficacy of UK best supportive care

Interviews with UK clinicians confirmed that the unusual long-term survival observed in a group of patients at the end of MDX010-20 could be a unique phenomenon of immunotherapy and should therefore be taken into account in the survival projections. The modelled survival projections were considered to be reasonably in line with clinical expectations.

Compared to the analysis results of Schadendorf *et al* for all patients receiving ipilimumab (Fig 1D), the model slightly under-predicts the long-term survival of patients receiving ipilimumab. However, this comparison should be treated with some caution as the analysis by Schadendorf *et al* contains a mix of treatment lines and observational and trial evidence. Compared to the Prieto protocol, short-term survival is lower than would be expected with similar long-term outcomes (Fig 1D) [16, 17].

Projected survival for BSC is comparable to observed evidence from the UK in second-line (1-year and 2-year survival rates of 28·1% and 13·8% versus 25.3% and 12.1%) [6].

## Discussion

The within-trial benefit from ipilimumab in MDX010-20 is large considering that, prior to this trial, no therapy for unresectable melanoma had demonstrated a significant survival benefit for 30 years. Within the trial, the increase in median survival over gp100 is 0.30 years, and the increase in mean survival is 0.54 years. The survival gains are more profound when trial results are modelled over a lifetime, with an expected mean survival of 2.77 years for ipilimumab and 1.07 years for gp100 (used as a proxy for BSC), leading to an expected gain in mean survival of 1.70 years per patient. The modelled long-term survival from MDX010-20 is comparable to available observed data for ipilimumab and the expectations of UK clinicians, indicating good external validity.

Based on the expected survival gains presented in this analysis, NICE recommended ipilimumab as a treatment option for patients with previously treated advanced (unresectable or metastatic) melanoma with an expected incremental cost-effectiveness ratio (ICER) of £42,200 per quality-adjusted life year (QALY) gained with the approved patient access scheme [9].

In July 2014 NICE extended this recommendation to patients at first-line, concluding that, without effective new therapies, the prognosis for advanced disease is very poor.[8] Clinical specialists confirmed to NICE that, for patients with BRAF V600 mutation-negative melanoma, dacarbazine was previously the only first-line treatment option available. Dacarbazine has never demonstrated a survival benefit in clinical trials and is associated with significant toxicity [7, 8]. In clinical practice, clinicians had to offer patients dacarbazine chemotherapy, with full knowledge of its low response rates and its associated high toxicity, before patients were eligible to be treated with second-line ipilimumab. For patients who have BRAF V600 mutation-positive melanoma, vemurafenib was the most widely used first-line treatment option (especially in those with a high disease burden). Vemurafenib offered high initial response rates but with limited duration of response. The vast majority of patients with BRAF mutant melanoma will have objective responses or temporary disease stabilization when treated with a BRAF inhibitor; however, most patients are likely to develop resistance to treatment and relapse within the first year of therapy (median progression-free survival is approximately 5–7 months for BRAF inhibitors) [24, 25].

The subsequent recommendation of ipilimumab as a first-line option offered additional choice for patients, particularly those with smaller-volume, more indolent disease. BRAF targeting agents could now be reserved as a rescue treatment later in the clinical pathway when their well-recognised rapid response may be more valuable. This is in line with recently published literature on treatment sequencing, which supports the sequencing of immunotherapy prior to BRAF inhibitors for less aggressive tumours [12, 13].

To understand the full benefit of treatment (including patients still alive at the end of trials), and to meet reimbursement requirements, the accurate estimation of the benefit of any treatment (in this case survival benefit) is key. Models, such as the one presented, can be used to extrapolate beyond the trial to final endpoints such as survival. Even when a clinical trial incorporates reasonably long follow-up, as is the case with MDX010-20, there may still be a need to extrapolate [26]. The duration of MDX010-20 was extremely long compared to other melanoma trials; however, at the end of the trial, a large proportion of patients remained alive. Patients were followed for up to 4.6 years in MDX010-20, with median follow-up times of 1.8 years in the ipilimumab-plus-gp100 group, 2.3 years in the ipilimumab-alone group, and 1.4 years in the gp100-alone group [14]. The need for accurate extrapolation of outcomes is even greater for other recent melanoma trials. For vemurafenib in the BRIM-3 trial, there was a maximum follow-up of 0.8 years; the median follow-up in the interim analysis was 0.3 years for patients in the vemurafenib group and 0.2 years for patients in the dacarbazine group, with a maximum of 2.8 years (median of 1.1 and 0.8 years, respectively) in the final published data cut. Similar (short) follow-up was observed for dabrafenib in BREAK-3 [27–29].

On the horizon are various new products for advanced melanoma, including the PD-1 blockade treatments nivolumab and pembrolizumab, and BRAF/MEK combination therapies. The ability to accurately project the long-term survival of patients receiving each of these therapies will be crucial in determining the comparative efficacy and value of new agents.

In conclusion, ipilimumab has been shown to be effective in the treatment of advanced melanoma, adding approximately 1.7 life years (2.8 vs 1.1) over the previous UK standard of care. This profound impact on survival has been reflected in reimbursement decisions both within the UK and worldwide. At the time of launch, ipilimumab represented a step change in the treatment of melanoma [7]. In the short time since, insights into the mechanisms of immune tolerance have guided researchers from the challenge of finding active drugs to the current questions of the optimum patient selection, timing, sequencing and combination of these therapies [30]. As more treatments emerge, the need for further research on appropriate sequencing will increase. Understanding the impact of new therapies on long-term prognosis via the use of appropriate modelling techniques will be vital to ensure that optimal outcomes are delivered for both patients and health care systems.

## References

[1] Cancer Research UK. Skin cancer: cancer statistics report 2013 [8 December 2014]. Available: http://publications.cancerresearchuk.org/downloads/Product/CS_CS_SKIN.pdf.

[2] Miller AJ, Mihm MC, Melanoma Jr.. The New England journal of medicine. 2006;355(1):51–65. Epub 2006/07/11. 10.1056/NEJMra052166 16822996

[3] Gogas HJ, Kirkwood JM, Sondak VK. Chemotherapy for metastatic melanoma: time for a change? Cancer. 2007;109(3):455–64. Epub 2007/01/04. 10.1002/cncr.22427 . 17200963

[4] Balch CM, Buzaid AC, Soong SJ, Atkins MB, Cascinelli N, Coit DG, et al Final version of the American Joint Committee on Cancer staging system for cutaneous melanoma. Journal of clinical oncology: official journal of the American Society of Clinical Oncology. 2001;19(16):3635–48. Epub 2001/08/16. . 11504745 10.1200/JCO.2001.19.16.3635

[5] Collinson FJ, Marples M. UK survey of second line chemotherapy use for metastatic melanoma. National Cancer Research Institute Conference 2010.

[6] Lorigan P, Marples M, Harries M, Wagstaff J, Dalgleish AG, Osborne R, et al Treatment patterns, outcomes, and resource utilization of patients with metastatic melanoma in the U.K.: the MELODY study. The British journal of dermatology. 2014;170(1):87–95. Epub 2013/07/17. 10.1111/bjd.12503 . 23855404

[7] Korn EL, Liu PY, Lee SJ, Chapman JA, Niedzwiecki D, Suman VJ, et al Meta-analysis of Phase II cooperative group trials in metastatic Stage IV melanoma to determine progression-free and overall survival benchmarks for future Phase II trials. Journal of clinical oncology: official journal of the American Society of Clinical Oncology. 2008;26(4):527–34. 10.1200/JCO.2007.12.7837 . 18235113

[8] National Institute for Health and Care Excellence (NICE). TA319: Melanoma (stage III or IV)—ipilimumab: guidance 2014 [8 December 2014]. Available: http://www.nice.org.uk/guidance/TA319.

[9] National Institute for Health and Clinical Excellence (NICE). TA268: Melanoma (stage III or IV)—ipilimumab: guidance 2012 [updated 12 December4 July 2013]. Available: http://guidance.nice.org.uk/TA268/Guidance/pdf/English.

[10] National Institute for Health and Clinical Excellence (NICE). TA269: Melanoma (stage III or IV)—vemurafenib: guidance 2012 [updated 12 December4 July 2013]. Available: http://guidance.nice.org.uk/TA269/Guidance/pdf/English.

[11] National Institute for Health and Clinical Excellence (NICE). TA343: Melanoma (stage III or IV)—dabrafenib: guidance 2014 [updated 12 December4 July 2013]. Available: http://guidance.nice.org.uk/TA343/Guidance/pdf/English.

[12] Jang S, Atkins MB. Which drug, and when, for patients with BRAF-mutant melanoma? The Lancet Oncology. 2013;14(2):e60–e9. 10.1016/s1470-2045(12)70539-9 23369684

[13] Ascierto PA, Margolin K. Ipilimumab before BRAF inhibitor treatment may be more beneficial than vice versa for the majority of patients with advanced melanoma. Cancer. 2014;120(11):1617–9. 10.1002/cncr.28622. 10.1002/cncr.28622 24577788

[14] Hodi FS, O'Day SJ, McDermott DF, Weber RW, Sosman JA, Haanen JB, et al Improved survival with ipilimumab in patients with metastatic melanoma. The New England journal of medicine. 2010;363(8):711–23. Epub 2010/06/08. 10.1056/NEJMoa1003466 20525992 PMC3549297

[15] Rosenberg SA, Yang JC, Restifo NP. Cancer immunotherapy: moving beyond current vaccines. Nature medicine. 2004;10(9):909–15. 10.1038/nm1100 15340416 PMC1435696

[16] Prieto PA, Yang JC, Sherry RM, Hughes MS, Kammula US, White DE, et al CTLA-4 blockade with ipilimumab: long-term follow-up of 177 patients with metastatic melanoma. Clinical cancer research: an official journal of the American Association for Cancer Research. 2012;18(7):2039–47. Epub 2012/01/25. 10.1158/1078-0432.ccr-11-1823 ; PubMed Central PMCID: PMCPmc3319861. 22271879 PMC3319861

[17] Schadendorf D, Hodi FS, Robert C, Weber JS, Margolin K, Hamid O, et al Pooled analysis of long-term survival data from Phase II and Phase III trials of ipilimumab in unresectable or metastatic melanoma. Journal of clinical oncology: official journal of the American Society of Clinical Oncology. 2015 Epub 2015/02/11. 10.1200/jco.2014.56.2736 . 25667295 PMC5089162

[18] Livingstone E, Zimmer L, Piel S, Schadendorf D. PLX4032: does it keep its promise for metastatic melanoma treatment? Expert opinion on investigational drugs. 2010;19(11):1439–49. Epub 2010/10/15. 10.1517/13543784.2010.527945 . 20942773

[19] Salama AK, Hodi FS. Cytotoxic T-lymphocyte-associated antigen-4. Clinical cancer research: an official journal of the A merican Association for Cancer Research. 2011;17(14):4622–8. Epub 2011/04/07. 10.1158/1078-0432.ccr-10-2232 . 21467163

[20] Latimer N. NICE DSU Technical Support Document 14: Survival analysis for economic evaluations alongside clinical trials- extrapolation with patient-level data 2013 [updated 27 February 2015]. Available: http://www.nicedsu.org.uk/NICE%20DSU%20TSD%20Survival%20analysis.updated%20March%202013.v2.pdf. 27905716

[21] Kotapati S, Dequen P, Ouwens M, van Baardewijk M, Ibrahim RA, Wagner S, et al. Overall survival (OS) in the management of pretreated patients with unresectable stage III/IV melanoma: A systematic literature review and meta-analysis. American Society of Clinical Oncology 47th Annual Meeting. Chicago, IL: USA; 2011.

[22] Annemans L, Asukai Y, Barzey V, Kotapati S, Lees M, van Baardewijk M, et al Extrapolation in oncology modelling: novel methods for novel compounds. ISPOR Connections. 2012;18(4):5–8.

[23] Office for National Statistics. Interim Life Tables, England, 1980–82 to 2007–09. 2010.

[24] Johnson DB, Sosman JA. Update on the targeted therapy of melanoma. Current treatment options in oncology. 2013;14(2):280–92. Epub 2013/02/20. 10.1007/s11864-013-0226-8 . 23420410 PMC6684217

[25] McArthur GA, Chapman PB, Robert C, Larkin J, Haanen JB, Dummer R, et al Safety and efficacy of vemurafenib in BRAF(V600E) and BRAF(V600K) mutation-positive melanoma (BRIM-3): extended follow-up of a phase 3, randomised, open-label study. The Lancet Oncology. 2014;15(3):323–32. Epub 2014/02/11. 10.1016/s1470-2045(14)70012-9 . 24508103 PMC4382632

[26] Buxton MJ, Drummond MF, Van Hout BA, Prince RL, Sheldon TA, Szucs T, et al Modelling in economic evaluation: an unavoidable fact of life. Health economics. 1997;6(3):217–27. Epub 1997/05/01. . 9226140 10.1002/(sici)1099-1050(199705)6:3<217::aid-hec267>3.0.co;2-w

[27] Hauschild A, Grob JJ, Demidov LV, Jouary T, Gutzmer R, Millward M, et al Dabrafenib in BRAF-mutated metastatic melanoma: a multicentre, open-label, Phase 3 randomised controlled trial. Lancet. 2012;380(9839):358–65. Epub 2012/06/28. 10.1016/s0140-6736(12)60868-x . 22735384

[28] Chapman PB, Hauschild A, Robert C, Haanen JB, Ascierto P, Larkin J, et al Improved survival with vemurafenib in melanoma with BRAF V600E mutation. The New England journal of medicine. 2011;364(26):2507–16. Epub 2011/06/07. 10.1056/NEJMoa1103782 ; PubMed Central PMCID: PMCPmc3549296. 21639808 PMC3549296

[29] Hauschild A, McArthur G, Robert C, Larkin J, Haanen JB, Ribas A, et al. Vemurafenib improves overall survival compared with dacarbazine in advanced BRAF v600-mutated melanoma: updated results from a Phase 3 randomized, multicenter trial. 10th International Meeting of the Society for Melanoma Research. Philadelphia, PA: USA; 2013.

[30] Bhatia S, Thompson JA. Melanoma: immune checkpoint blockade story gets better. Lancet. 2014;384(9948):1078–9. 10.1016/S0140-6736(14)61140-5 . 25034863

